# The Use of Urea and Kelp Waste Extract is A Promising Strategy for Maximizing the Biomass Productivity and Lipid Content in *Chlorella sorokiniana*

**DOI:** 10.3390/plants9040463

**Published:** 2020-04-07

**Authors:** Ali Nawaz Kumbhar, Meilin He, Abdul Razzaque Rajper, Khalil Ahmed Memon, Muhammad Rizwan, Mostafa Nagi, Abeselom Ghirmai Woldemicael, Dan Li, Chun Wang, Changhai Wang

**Affiliations:** 1 Jiangsu Provincial Key Laboratory of Marine Biology, College of Resources and Environmental Sciences, Nanjing Agricultural University, Nanjing 210095, China; alinawazapt@yahoo.com (A.N.K.); hemeilin@njau.edu.cn (M.H.); razarajper6@gmail.com (A.R.R.); khaleelmemon27@gmail.com (K.A.M.); mostafa.n.bakri@gmail.com (M.N.); 2017103207@njau.edu.cn (A.G.W.); 2018203016@njau.edu.cn (D.L.); 2019203008@njau.edu.cn (C.W.); 2 US Pakistan Center for Advanced Studies in Water, Mehran University of Engineering and Technology; Jamshoro 76062, Pakistan; drmrizwan.uspcasw@faculty.muet.edu.pk

**Keywords:** urea, kelp waste extracts, biomass, lipid, biofuel, *Chlorella sorokiniana*

## Abstract

The decline in fossil fuel reserves has forced researchers to seek out alternatives to fossil fuels. Microalgae are considered to be a promising feedstock for sustainable biofuel production. Previous studies have shown that urea is an important nitrogen source for cell growth and the lipid production of microalgae. The present study investigated the effect of different concentrations of urea combined with kelp waste extract on the biomass and lipid content of *Chlorella sorokiniana.* The results revealed that the highest cell density, 20.36 × 10^7^ cells^−1^, and maximal dry biomass, 1.70 g/L, were achieved in the presence of 0.5 g/L of urea combined with 8% kelp waste extract. Similarly, the maximum chlorophyll a, b and beta carotenoid were 10.36 mg/L, 7.05, and 3.01 mg/L, respectively. The highest quantity of carbohydrate content, 290.51 µg/mL, was achieved in the presence of 0.2 g/L of urea and 8% kelp waste extract. The highest fluorescence intensity, 40.05 × 10^7^ cells^−1^, and maximum total lipid content (30%) were achieved in the presence of 0.1 g/L of urea and 8% kelp waste extract. The current study suggests that the combination of urea and kelp waste extract is the best strategy to enhance the biomass and lipid content in *Chlorella sorokiniana*.

## 1. Introduction

Recent reports have suggested that the world fossil fuel reserves are likely to be exhausted in less than 45 years due to the fast-growth of transportation and industries [1]. The increasing price of fossil fuels is a serious problem for countries with limited resources [2]. Governments and private sectors are actively engaged in determining substitutes for fossil fuel energy [2]. There are different forms of alternative energy, such as wind, solar, hydroelectric, nuclear, and biofuel, which have been introduced as substitutes for fossil fuel. Recently, biofuel energy has been receiving the interest of researchers and international traders due to it being a clean and sustainable source of energy [3]. Biofuels are chemically defined as the monoalkyl esters of long-fatty acid (FA) chains derived from renewable feedstocks, such as soybean oil, palm oil, rapeseed oil, and sunflower [4,5]. However, these crops need extensive water and land for cultivation; therefore, the productive and cultivated land must be used for food instead of biofuel production [6]. 

Microalgae are an extremely promising biofuel feedstock due to the following reasons: microalgae have higher photosynthetic efficiencies, a rapid growth rate (commonly doubling its biomass within 24 h), a high lipid content compared to terrestrial crops [7], tolerance to extreme conditions (desert and arid lands) [8], a lesser impact on the environment and on the global food supply, less need of land for cultivation [9,10], and, finally, the production of substantial biomass (10–40 g DW m^−2^ day^−1^) per unit land area, producing as much as two times to ten times more biomass than terrestrial crops [11,12]. The lipid content of microalgae is usually in the range of 20–50% [7]. The lipid percentages of different microalgae assumed by various scholars are shown in Table 1.

To date, more than 40,000 species of microalgae serve as raw materials for several industries, including biofuel production [16]. Among the all green microalgae species, the genus *Chlorella* have the ability to grow under various stress conditions [17]. Compared with other *Chlorella* species, the *Chlorella sorokiniana* is known to be a promising candidate for biofuel production due to sufficient (18–22%) lipid content [17,18,19]. Unfortunately, the biofuel production from this microalgae is currently not economically competitive with fossil fuels due to high operational costs [20]. The production costs have been estimated at approximately US 470/ton [7]. 

The most essential nutrients (nitrates, phosphates, and glucose) that contribute to the production of biomass and lipid content are costly for large scale cultivation. Several strategies were applied to reduce the cultivation cost, such as nitrogen starvation and wastewater cultivation [21,22]. However, these strategies failed to increase the biomass yield and lipid contents at a large scale [23]. The kelp waste is the residue after extracting alginate from kelp (Aresch), which contains a large amount of macroelements and trace elements. These could be used to stimulate the growth of plants and microbes [24]. In addition, most of these solid wastes were released directly in landfills, resulting in a severe waste of resources and the occupation of a great deal of land [25]. 

Thus, the recycling of kelp waste provides great potential for the reduction of waste pollution and improvement of large-scale microalgae cultures for biofuel production. Earlier research demonstrated that the presence of a nitrogen source in the cultivation media significantly affected both the growth rate and oil content of the algae [26]. Urea is a cheap nitrogen source that is commonly used for microalgae cultivation due to its universal availability and affordability [27,28]. A previous study demonstrated that the 8% kelp waste extract (KWE) improved the growth rate and lipid content of *Chlorella sorokiniana* [29]. To our knowledge, there is no recent study using kelp waste extract combined with urea to maximize the biomass and lipid content of *Chlorella sorokiniana*. This study investigated the effects of different concentrations of urea combined with kelp waste extract on the growth and biochemical composition of *Chlorella sorokiniana.*

## 2. Results

### 2.1. Effect of Urea and KWE on the Cell Density of C. sorokiniana 

**Figure 1:**
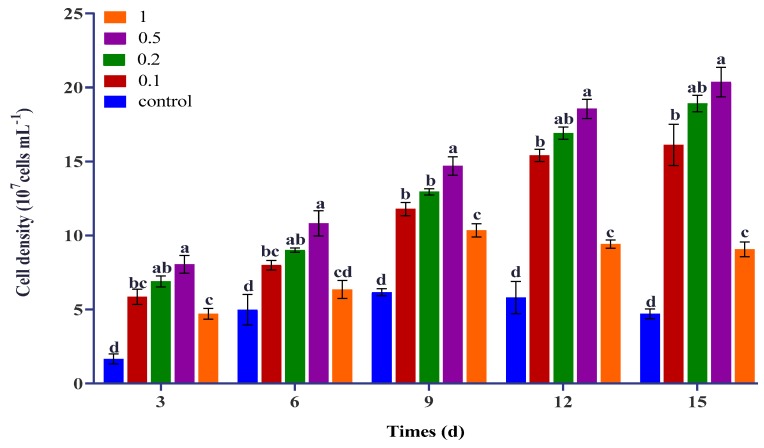
Effect of different concentrations of urea combined with kelp waste extract (KWE) on the cell density of *C. sorokiniana*. The bar charts represent the means ± SD of triplicate samples (*n* = 3). Different alphabets letters indicate significant differences (*p* < 0.05) between the urea concentrations.

The changes in the cellular density of *C. sorokiniana* under different concentrations of urea (0.1, 0.2, 0.5, and 1 g/L) combined with 8% KWE during 15 days of incubation are illustrated in Figure 1. We observed that, until the second day of the experiment, the cells adjusted themselves in the modified medium. From the third day until day 15, the culture supply with an intermediate concentration of urea and KWE showed rapid cell growth. However, the cultures supplied with a higher concentration of urea decreased in cell density after 9 days of cultivation. A similar trend was observed in the control medium; the cell density decreased after 9 days of cultivation due to the lack of nutrients. The cells grew in the presence of 0.1 and 0.2 g/L of urea combined with 8% KWE increased cell density to 16.76 × 10^7^ and 18.90 × 10^7^ cells mL^−1^, respectively. In comparison, the cells supplied with 0.5 g/L of urea combined with 8% KWE produced the highest (20.36 × 10^7^ cells mL^−1^) density whereas the cells that grew in the regular Bold’s basal medium (BBM) medium showed a fourfold lower cell density as compared to the highest cell density.

### 2.2. Effect of Urea and KWE on the Chlorophyll Pigments of C. sorokiniana 

**Figure 2:**
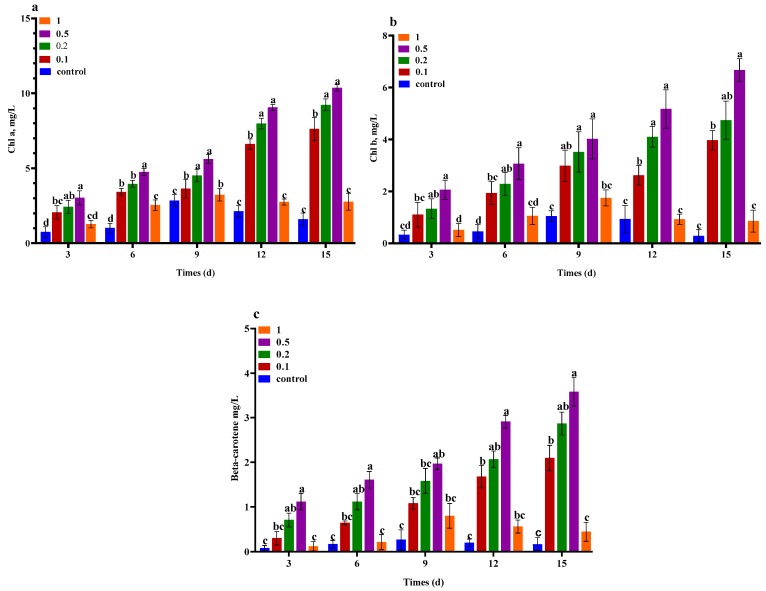
Content in chlorophyll (**a**) and (**b**) and carotenoids in (**c**). *C. sorokiniana* grown under the different concentrations of urea combined with 8% KWE. The bar charts represent the means ± SD of triplicate samples (*n* = 3). Different alphabets letters indicate significant differences (*p* < 0.05) between the urea concentrations.

The chlorophyll content showed slow growth in all urea concentrations until 6 days. After 6 days, a stronger increase was found in the chlorophyll content until 15 days. In the case of the control medium, the pigment content increased until 9 days and then slightly decreased until 15 days. Figure 2a–c shows the effect of different concentrations of urea combined with KWE on the pigment contents of *C. sorokiniana*. The maximum values of chlorophyll a at 10.36, b at 7.05, and beta carotene at 3.01 mg/L were recorded in the presence of 0.5 g/L of urea combined with 8% KWE. While the second number highest value of chlorophyll a at 9.07, b at 4.73, and beta carotene at 2.39 mg/L were recorded in the presence of 0.2 g/L of urea combined with 8% KWE whereas the cells supplied with a higher concentration of urea at 1 g/L combined with 8% KWE increased in chlorophyll content until 9 days and then continuously decreased in chlorophyll until 15 days. However, the cells grown in the control medium showed less accumulations with chlorophyll a at 1.79, b at 1.01, and beta carotene at 0.45 mg/L content.

### 2.3. Effects of Urea and KWE on the Carbohydrate Content of C. sorokiniana 

**Figure 3:**
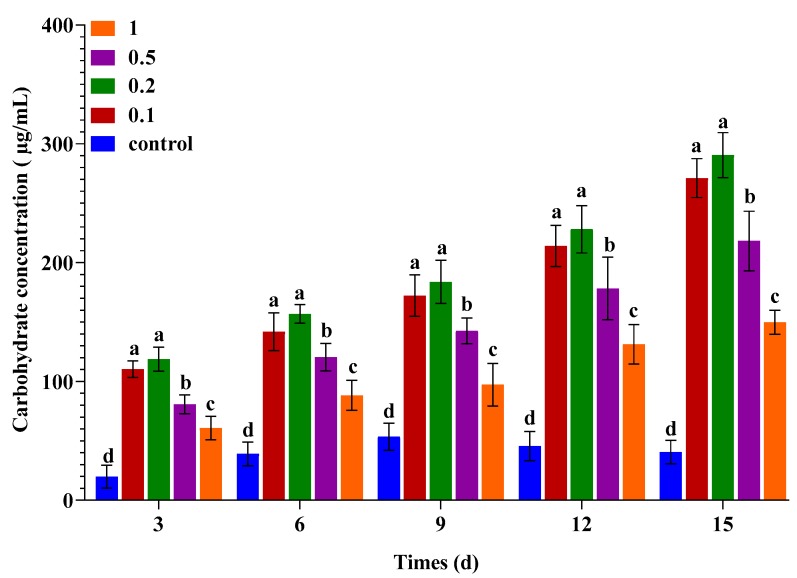
Carbohydrate content of *C. sorokiniana* during the cultivation in different concentration of urea combined with KWE. The bar charts represent the means ± SD of triplicate samples (*n* = 3). Different alphabets letters indicate significant differences (*p* < 0.05) between the urea concentrations.

The effects of different concentrations of urea and KWE on the carbohydrate content of *C. sorokiniana* are shown in Figure 3. The results indicate that the low concentration of urea combined with 8% KWE enhanced the carbohydrate content. The culture contained 0.1 and 0.2 g/L of urea combined with 8% KWE, and their values were slightly different from each other, from day 3 to day 15 respectively. Among all urea concentrations, the cells cultured in 0.5 g/L and 1 g/L of urea increased their carbohydrate content by a low amount. In general, the highest carbohydrate of 290.51 µg/mL was achieved in the presence of 0.2 g/L of urea and 8% KWE whereas the lowest value of carbohydrate content 40.55 µg/mL, was recorded in the control culture. The results of our present study suggest that the addition of 0.2 g/L of urea combined with 8% KWE is the best strategy to accumulate the carbohydrate content of *C. sorokiniana*.

### 2.4. Effect of Urea and KWE on the Neutral Lipid of C. sorokiniana

**Figure 4:**
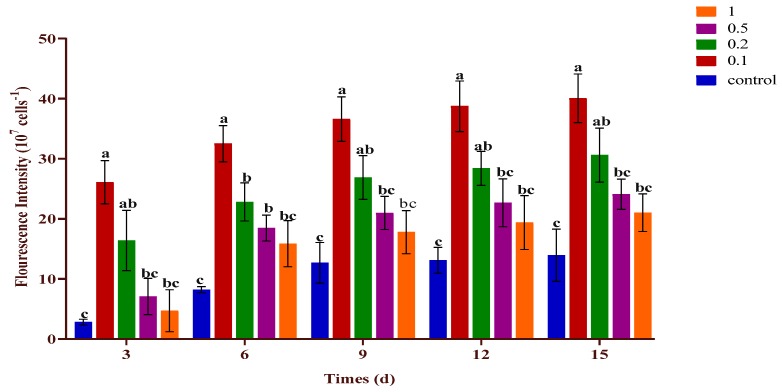
Nile red fluorescence intensity of *C. sorokiniana* (expressed as 10^7^ cells mL^−1^) under different concentrations of urea combined with KWE. The bar charts represent the means ± SD of triplicate samples (*n* = 3). Different alphabets letters indicate significant differences (*p* < 0.05) between the urea concentrations.

*C. sorokiniana* was cultivated for 15 days under different concentrations of urea (0.1–1 g/L) combined with 8% KWE to determine the best level of urea combined with KWE to maximize the neutral lipid content. Figure 4 displays the effects of different concentrations of urea and 8% KWE on the neutral lipid content of *C. sorokiniana*. The highest fluorescence intensity was recorded under urea regime, which was 0.1 g/L and 0.2 g/L of urea and 8% KWE. A decreasing trend in fluorescence intensity was recorded under the higher concentrations of 0.5 and 1 g/L of urea and 8% KWE. The high regime of 1 g/L of urea combined with 8% KWE significantly decreased the fluorescence intensity. The highest fluorescence intensity of 40.05 × 10^7^ cell^−1^ was recorded under the 0.1 g/L of urea and 8% KWE regime. A lower intensity of 13.94 × 10^7^ cell^−1^ was recorded for the control culture.

### 2.5. Effect of Urea and KWE on the Biomass and Lipid Contents of C. sorokiniana

**Figure 5:**
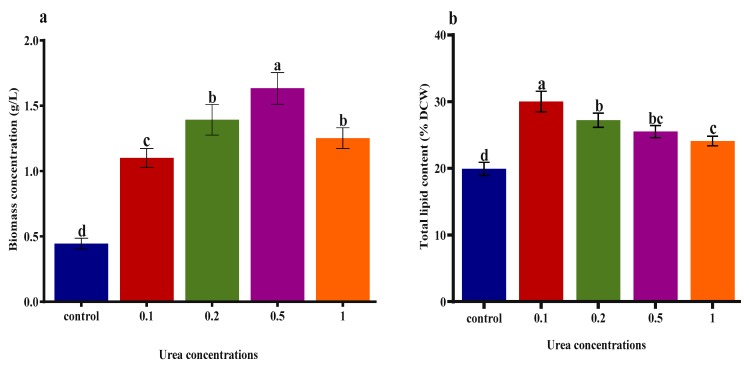
(**a**) Biomass and (**b**) lipid content of *C. sorokiniana* under different concentrations of urea combined with KWE. The bar charts represent means ± SD of triplicate samples (*n* = 3). Different alphabets letters indicate significant differences (*p* < 0.05) between urea concentrations and control.

Figure 5a illustrates the effect of urea and KWE on the biomass concentrations of *C. sorokiniana.* Different concentrations of urea combined with KWE were applied to maximize the biomass concentration. According to the results in Figure 5a, the higher concentration of urea at 1 g/L and 8% KWE decreased the cell growth of algae, which resulted in a lower biomass concentration. The result showed that the maximum biomass concentration of 1.70 g/L was achieved at 0.5 g/L of urea combined with 8% KWE, and the lowest 0.44 g/L concentration of biomass was found in the control culture. Figure 5b demonstrates the effect of urea and KWE on the lipid accumulation of *C. sorokiniana*. The results demonstrated that the total lipid content increased with a decreasing concentration of urea. The cells cultivated in the higher concentrations of 0.5 and 1 g/L of urea combined with 8% KWE produced total lipid contents of 25% and 24%, respectively. In contrast, the cells cultivated at the lower concentrations of 0.1 and 0.2 g/L of urea combined with 8% KWE produced total lipid contents of 30% and 27%, respectively. The culture cultivated in the regular BBM medium produced a total lipid content of 19%. 

## 3. Discussion

Nitrogen is an essential nutrient, and the type of nitrogen has a significant effect on the efficiency of cell growth and the metabolite formation of microalgae [30]. Several studies have reported that the biochemical composition of microalgae can be affected by modifications to the culture media, such as nitrogen limitations [27,31]. Urea is the most suitable nitrogen source for algal growth as it is less expensive compared to other nitrogen sources, and this would be a major advantage for industrial processes [32]. However, relatively scarce information is present on the use of urea combined with waste substances to maximize the biomass and lipid content of *C. sorokiniana.*

In the present study, urea was used as a nitrogen source combined with kelp waste extract as a multifunctional biostimulant to enhance the cell growth and lipid content of *C. sorokiniana.* The results indicate that the intermediate concentration of 0.5 g/L of urea combined with 8% KWE increased the cell growth. The increasing concentration (≥0.5 g/L) of urea led to a decrease in cell growth due to the ammonium toxicity from the growth medium due to the high concentration of urea. Urea quickly breaks into ammonium and CO_2_; therefore, the higher concentration of ammonium in the growth medium caused a decrease in the pH, which can destroy the cell photosynthesis process and result in mortality of the cell [33]. 

Meanwhile, the highest cell density, 20.36 × 10^7^ cells mL^−1^, was recorded in the presence of 0.5 g/L of urea combined with 8% KWE. In fact, the cell density recorded in the present study was six times higher than the density (3.57 × 10^8^ cells mL^−1^) of *Chlorella* sp. E1708 as reported in a previous study [33]. Mulders [34] stated that the presence of a higher concentration of nitrogen in the growth medium tended to increase the chlorophyll content of *Chlorella zofingiensis*. However, in the current study, an inverse relationship between the increasing concentration of urea and chlorophyll content was observed. The lowest values of chlorophyll a and b and beta carotene were observed at a higher concentration of urea combined with KWE, due to inhibition of the substrate, which blocks the chlorophylls synthesis in algal cells [35]. 

Compared to all urea concentrations, the highest value of chlorophyll content was recorded at the intermediate concentration of 0.5 g/L of urea combined with 8% KWE. This enhancement in chlorophyll was observed due to the presence of KWE (C, N, P, and Mg) nutrients. However, there is no carbon source in Bold’s basal medium, which consists of N, P, potassium (K), calcium (Ca), magnesium (Mg), and other essential trace elements to enhance microalgae growth. In the regular Bold’s basal medium, the N and P contents were significantly lower than the KWE nutrients. Similar results of increasing in chlorophyll content were also reported by [36] who observed that the addition of FeSO_4_ and MgSO_4_ combined with urea in cultivation medium significantly increased the chlorophyll a and b and carotenoid pigment contents of *C. vulgaris*. 

In previous studies it was discussed that microalgae alter their biomass composition under stress conditions to accumulate carbohydrate content [37]. In the present study, the KWE nutrients showed excellent performance in carbohydrate enhancement. According to the results, the carbohydrate contents were higher at lower concentrations (0.1 and 0.2 g/L) of urea than at higher concentrations (0.5 and 1 g/L). This result is in agreement with the literature suggesting [38] that under the N deficient condition, N containing macromolecules and carbon reserve compounds like carbohydrates are increased. Another researcher [39] also observed accumulation in the carbohydrate content of *Scenedesmus* sp. CCNM 1077 under nitrogen starvation conditions. 

A number of studies reported that nitrogen starvation increased the accumulation of lipid content in microalgae [40,41]. Du and Benning [42] reported that numerous microalgae species under nitrogen stress changed their biosynthetic pathways toward the accumulation of total lipids and neutral lipids. Similar results were observed in our study, where the minimal urea supply strategy combined with KWE for *C. sorokiniana* cultivation produced the maximum amount of total lipids and neutral lipids per cell as compared to the higher concentration of urea. 

Comparable results were confirmed by another study [43] in which the authors observed that the addition of a minimal concentration of urea maximized the lipid content of *Chlorella* sp. A2 compared to the regular BG11 medium. According to the results achieved in the present study, the highest 1.70 g/L biomass concentration (on a dry weight basis) was achieved under the 0.5 g/L concentration of urea. A further increase in the urea concentration (>0.5 g/L) led to a decrease in the biomass content. 

The present study results are in agreement with a study [44] where the authors reported that the lower concentration of 0.5 g/L of urea gave the highest (6.7 g/L) biomass of *Chlorella pyrenoidosa.* The higher concentration of urea (>0.5 gL^−1^) led to a decrease in the biomass content. Based on the above results, it was clear that the lipid content and the biomass yield of *C. sorokiniana* were higher in the modified medium than those of the regular BBM medium. Furthermore, the kelp waste is a large industrial residual from alginate processing in China, and feedstock of kelp waste is easily available in China. The KWE one liter production cost is 0.28 USD based on the price of the enzymes used [45]. Finally, the utilization of urea combined with KWE could reduce the cost on a large scale.

## 4. Materials and Methods 

### 4.1. Microalgae Source and Seed Culture 

The microalgae *Chlorella sorokiniana* (FACHB-275) was obtained from the Institute of Hydrobiology, Chinese Academy of Sciences. The culture was prepared in Bold’s basal medium (BBM) [46]. The culture volume was 100 mL in a 250 mL flask. The cells were incubated at room temperature (25 ± 1 °C). The flasks were placed in front of cool-white fluorescent lamps (45 μmol m^−2^ s^−1^) with 14 h light/10 h dark periods. 

### 4.2. Experimental Details 

The study was conducted in the laboratory of Marine Science College of Resource and Environment Science Nanjing Agriculture University China. Kelp waste extract (KWE) was prepared with enzymolysis as reported by the authors in [29]. The composition of BBM and KWE [29] is shown in Table 2. In order to investigate the effect of KWE and urea on the cell growth and biochemical composition of *C. sorokiniana*, the cells were incubated with 8% (*v/v*) kelp waste extract combined with different concentrations of urea (0.1, 0.2, 0.5, and 1 g/L). The original BMM medium was considered as the control. The culture flasks were incubated at 25 °C (±1 °C) with cool fluorescent light at 45 μmol m^−2^ s^−1^ and a 16:8 light/dark period ratio. The flasks were manually shaken gently three times a day to prevent sticking of the algal cells to the walls of the flasks. All the treatments and the control flask had three replicates and the experiment was run for 15 days. 

### 4.3. Measurements of Cellular Density and Chlorophyll Pigments 

The microalgae growth was observed using the optical density (OD) at a wavelength of 750 nm with a SpectraMax M5 microplate reader (Molecular Devices, US). The chlorophyll content was measured according to a previously described process [47]. The 10 mL of algal culture inserted into a centrifuge tube were then centrifuged at a speed of 8000 rpm for 10 min, and then 2 mL of methanol was added to the pellet and kept at 4 °C for 24 h in the dark. After 24 h the sample was transferred into a spectrophotometer glass cuvette and the chlorophyll a and b and beta-carotene contents were measured at 470, 653, and 666 nm, respectively. Finally, the relative amounts of pigments were calculated by the following equations and expressed in mg/L.

Chlorophyll a (Chl a) = 15.65A666 − 7.34A653

Chlorophyll b (Chl b) = 27.05A653 − 11.21A666

Beta-Carotene (Bc) = (1000A470 − 2.86Ca − 129.2Clb)/245.

### 4.4. Carbohydrate Quantification 

The carbohydrate contents were determined through the standard method [48]. We centrifuged 5 mL of the algal culture at 8000 rpm for 10 min. The pellet was added into 0.5% phenol in 1 mL of H_2_SO_4_ then the mixture was incubated at 37 °C for 30 min in a waterbath. Finally, the carbohydrate contents were recorded spectrometrically at 490 nm.

### 4.5. Measurement of Neutral Lipid Contents 

The neutral lipid content was determined using Nile red staining [49]. We mixed 1 mL of culture in 330 μL of 25% dimethyl sulfoxide and then sonicated for 1 min by ultrasonication (KQ5200B, China). Then, 15 μL of Nile red (0.1 mg mL^−1^ acetone) was added into the mixture; the cells were stained at 40 °C for 10 min in a water bath. The fluorescence intensity was measured by a SpectraMax M5 Microplate Reader with the wavelengths of excitation and emission at 480 nm and 575 nm, respectively. 

### 4.6. Estimation of the Dry Cell Weight and Total Lipid Contents

In order to extract the dry biomass weight of the culture, the algal cells were collected at a stationary phase by centrifugation at 4 °C and 6800× *g* for 10 min and then dried by a lyophilizer for 48 h. After obtaining the constant weight, the weight of centrifuge tube was subtracted from the weight before drying. The biomass concentration was expressed in g/L. The content of the total lipids was measured with chloroform methanol based on a modified method [50]. The procedure was as described by [29]. The lipid content was measured gravimetrically and expressed as the dry cell weight percentage (%DCW).

### 4.7. Statistical Analysis

All experiments were conducted in triplicate. The data were statically analyzed by Graph Pad Prism, version 8.0. All values were expressed as the mean ± SEM. The statistical differences were assessed by two-way ANOVA considering Bonferroni post-tests to compare the means of the replicates, where *P*-values < 0.05 were considered significant.

## 5. Conclusions 

*Chlorella sorokiniana* is promising species for biofuel production, and therefore maximizing the cultivation process of *Chlorella sorokiniana* is useful. The present study results demonstrated that *Chlorella sorokiniana* has the ability to maximize the biomass and lipid content under a low level of urea combined with KWE. The optimal biomass was achieved in the presence of 0.5 g/L urea and 8% KW. In addition, the maximum total lipid contents were achieved under the treatments of 0.1 g/L and 8% KWE. The combination of urea and KWE also showed the best enhancements in the chlorophyll, neutral lipid, and carbohydrate contents. The current study suggests that the strategy of KWE combined with urea provided a novel idea for sustainable algal fuel at a large level.

## Figures and Tables

**Table 1:** Lipid content of different algal species [7,13,14,15].

Microalgae	Biomass Productivity (mg L^−^^1^ d^−^^1^)	Lipid Content (% of biomass)	Lipid Productivity (mg L^−^^1^ d^−^^1^)
*Chlorella sorokiniana*	315.5 ± 10.3	19.8 ± 0.7	62.3 ± 2.0
*Tetraselmis* sp. LW	414.0 ± 11.3	14.9 ± 0.1	61.8 ± 1.7
*Chlorella* sp. AMI2	307.3 ± 7.7	19.2 ± 0.4	59.0 ± 1.5
*Porphyridium cruentum*	613.3 ± 77	8 9.4 ± 0.2	57.5 ± 7.3
*Tetraselmis suecica* CV	383.6 ± 1.3	14.9 ± 0.1	57.3 ± 0.2
*Chlorella vulgaris* UTEX 1200	274.5 ± 21.9	19.4 ± 0.9	53.2 ± 4.2
*Monodus subterraneus* UTEX	257.3 ± 20.6	15.5 ± 0.5	39.9 ± 3.2
*Tetraselmis suecica* OR	448.0 ± 0.0	8.4 ± 0.3	37.5 ± 0.0

**Table 2:** Composition of Bold’s basal medium (BBM) and kelp waste extract (KWE).

BBM		KWE	
NaNO_3_ (mg L^−1^)	250.00	N (mg L^−1^)	5723.93 ± 75.21
MgSO_4_·7H_2_O (mg L^−1^)	75.00	P (mg L^−1^)	5529.45 ± 33.94
NaCl (mg L^−1^)	25.00	K (mg L^−1^)	60.54 ± 0.43
K_2_HPO_4_ (mg L^−1^)	75.00	Ca (mg L^−1^)	54.91 ± 4.51
KH_2_PO_4_ (mg L^−1^)	175.00	Mg (mg L^−1^)	75.64 ± 5.94
CaCl_2_·2H_2_O (mg L^−1^)	25.00	Fe (mg L^−1^)	ND
ZnSO_4_·7H_2_O (mg L^−1^)	8.82	Mn (mg L^−1^)	0.65 ± 0.06
MnCl_2_·4H_2_O (mg L^−1^)	1.44	Cu (mg L^−1^)	0.04 ± 0.09
MoO_3_ (mg L^−1^)	0.71	Zn (mg L^−1^)	8.30 ± 1.75
Co (NO_3_)_2_·6H_2_O (mg L^−1^)	0.49	B (mg L^−1^)	6.04 ± 0.85
H_3_BO_3_ (mg L^−1^)	11.42	Amino acids (mg L^−1^)	194.03 ± 0.75
EDTA (mg L^−1^)	50.00	Reducing sugars (g L^−1^)	19.55 ± 0.13
KOH (mg L^−1^)	31.00	Total sugars (g L^−1^)	23.19 ± 0.65
FeSO_4_·7H_2_O (mg L^−1^)	4.98	Alginic acid (g L^−1^)	6.09 ± 0.44
H_2_SO_4_ (conc., mL)	1.00		
